# Surgical Treatment of Adrenal Gland Metastasis Originating from Small Cell Carcinoma of the Urinary Bladder

**DOI:** 10.1155/2013/982787

**Published:** 2013-12-08

**Authors:** Minekatsu Taga, Hideaki Ito, Naoya Kusukawa, Yoshiji Miwa, Hironobu Akino, Yoshiaki Imamura, Osamu Yokoyama

**Affiliations:** ^1^Division of Urology, Department of Surgery, Faculty of Medical Sciences, University of Fukui, 23-3 Matsuoka Shimoaiduki, Eiheiji-cho, Yoshida-gun, Fukui 910-1193, Japan; ^2^Department of Urology, Fukui Social Insurance Hospital, Fukui 911-8558, Japan; ^3^Division of Surgical Pathology, University of Fukui Hospital, Fukui 910-1193, Japan

## Abstract

We report a rare case of a solitary adrenal metastasis from small cell carcinoma of the urinary bladder that was successfully treated with surgical resection. A 71-year-old man was suffering from bladder tamponade for hematuria. Computed tomography (CT) revealed a bladder tumor at the left wall. The patients underwent radical cystectomy. Histopathological results were obtained in small cell carcinoma of the bladder with muscle invasion. Thus, he received two courses of adjuvant etoposide and cisplatin chemotherapy, followed by the regimen for small cell lung cancer. Seven months after surgery, follow-up CT showed a gradually enlarged mass enhanced heterogeneously in the right adrenal gland. There was a solitary adrenal metastasis without any other metastasis; therefore, we performed right laparoscopic adrenalectomy. The patient has remained uneventful for four years after the adrenal gland surgery. For patients who have a solitary adrenal metastasis, adrenalectomy may provide a survival benefit.

## 1. Introduction

Small cell carcinoma of the bladder (SCCB) is exceedingly rare and accounts for less than 1% of all bladder carcinomas. The majority of patients are elderly, with a male : female ratio of 3 : 1, and they often have a history of smoking. The most common symptom was gross hematuria for 68.2% of patients [1]. Because SCCB is mostly diagnosed at an advanced stage and behaves aggressively, the prognosis of patients with SCCB is poor; overall survival at five years ranges from 8% to 25% [1–3].

SCCB is frequently managed by radical cystectomy with adjuvant chemotherapy, but there is no established treatment for the disease. It is also rare that bladder carcinoma can cause a solitary adrenal metastasis, but it has been reported that surgical resection could improve survival [4].

We present a case of laparoscopic adrenalectomy as a treatment for solitary adrenal metastasis from SCCB, which was identified even after cystectomy and two courses of adjuvant etoposide and cisplatin (EP) chemotherapy.

## 2. Case Presentation

**Figure 1:**
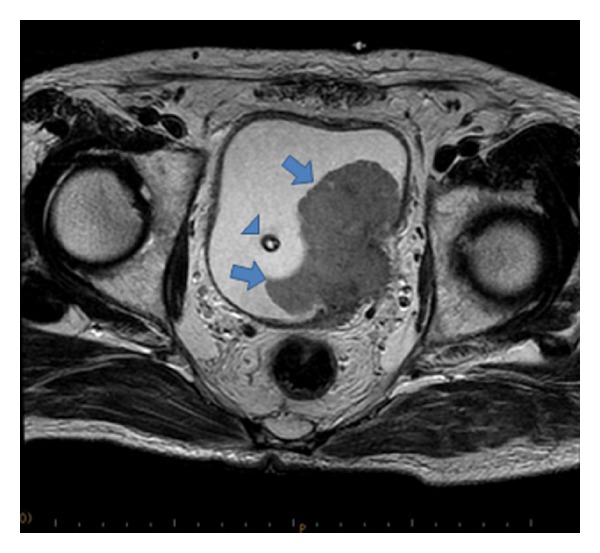
Enhanced MRI showing a bladder tumor at the left wall (arrow). Arrow head shows urethral balloon catheter.

A 71-year-old man was hospitalized for bladder bloody tamponade. Cystoscopy revealed a broad-based tumor at the left wall of the urinary bladder. Urinary cytologic findings indicated poorly differentiated urothelial carcinoma. A computed tomography (CT) scan showed a 7.0 cm bladder tumor at the left wall with a complicated left hydronephrosis (Figure 1). A magnetic resonance imaging (MRI) scan disclosed deep invasion into the muscle layers of the bladder and invasion of the left ureter. Preoperative tumor markers indicated elevation in NSE (16.0 ng/mL: normal range 0–12.0), but proGRP was normal at <10.0 pg/mL.

The patient underwent cystectomy. During surgery, we examined the distal urethral stump pathologically and performed ureteroscopy of the left ureter. These procedures showed no residual tumor in the upper urinary tract.

**Figure 2:**
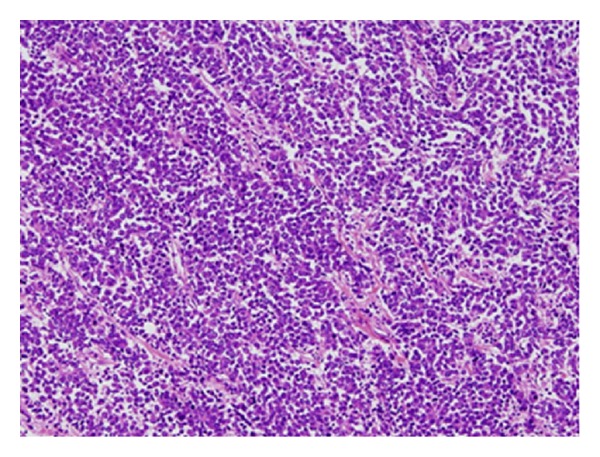
Histopathological examination of small cell carcinoma of the urinary bladder with H & E staining (×100). Tumor cells are characterized by a high nuclear to cytoplasmic ratio.

Postoperative histopathological results demonstrated SCCB with muscle invasion and vascular invasion, which admixed with the urothelial carcinoma only in a small part (Figure 2).

Tumor markers of NSE and proGRP decreased to be within normal limits. According to the classification of TNM, the clinical stage was T3N0M0. Consequently, the patient underwent postoperative chemotherapy with two courses of EP therapy according to the protocol for primary small cell lung cancer (etoposide 80 mg/m^2^ on days 1–3; cisplatin 100 mg/m^2^ on day 1).

**Figure 3:**
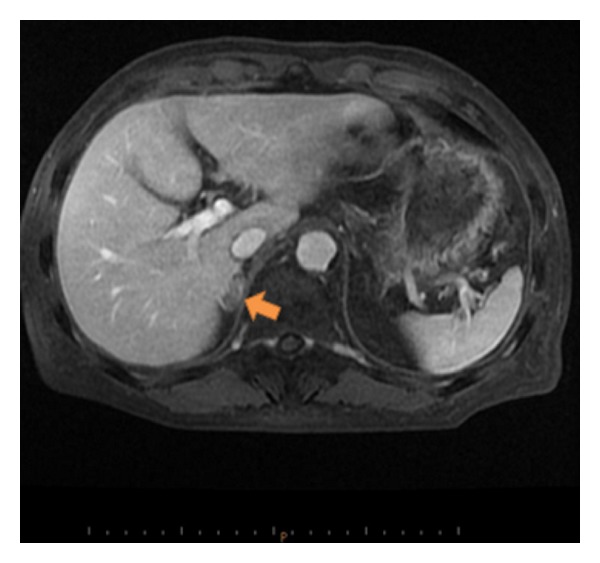
Surveillance-enhanced MRI scan showing enlargement of the right adrenal gland seven months after radical cystectomy.

Surveillance CT scan performed four months postoperatively detected a slight enlargement of the right adrenal gland, which had not been present in the previous scan. Three months later, follow-up CT-MRI scan showed a gradually progressing 2.5 × 1.5 cm mass. No other metastatic sites were identified. The adrenal mass was suspected to be either a primary adrenal cancer or a metastasis from the bladder because the mass had demonstrated heterogeneous enhancement (Figure 3).

Laboratory examination showed that tumor markers such as NSE and proGRP had remained within normal limits and showed elevation of the serum noradrenaline (1.12 ng/mL; normal range 0.10–0.50) and dopamine levels (0.04 ng/mL; 0–0.03), but MIBG scintigraphy displayed no uptakes in the right adrenal gland. Hence, we diagnosed the right adrenal metastasis as being from bladder cancer and performed laparoscopic right adrenalectomy nine months after the primary surgery. The histopathology of the adrenal specimens verified the character of the metastasis as being from SCCB. After the laparoscopic adrenalectomy, additional adjuvant chemotherapy was not undertaken by the refusal of the patient.

At the patient's ambulatory follow-up, the tumor markers, NSE and proGRP, remained within normal limits, and he has been uneventful for four years after laparoscopic adrenalectomy.

## 3. Discussion

Small cell carcinomas (SCC) most commonly appear in the lung, and it is unusual for these cells to arise in extrapulmonary sites. Blomjous et al. [5] reported that the prevalence of SCC as a primary urinary bladder malignancy was 0.48% in 3,778 cases. Most SCCB patients already have metastatic disease at the first visit, and even after surgery is performed once, many of them develop hematogenous or lymphatic metastasis early in the postoperative course. Therefore, the clinical outcome of SCCB is much worse than that of transitional cell carcinoma.

At the time of diagnosis, almost all of the SCCB present are at the advanced stage, and from 96% to 100% of them already show local invasion or metastatic sites [1, 3, 6].

The sites of metastasis from a primary SCCB presented in a lymph node in 28% to 53% of cases, the liver in 24% to 47%, and the bone in 23% to 33%, while lung metastasis, which is common in urothelial carcinoma, occurred in only 10% to 13% of cases [1, 6]. Abbas et al. [2] reported a two-year survival rate of all stages of 19.8% and a five-year rate of 8.1%.

Although there is no consensus on a standard therapy, combined modalities of therapy have been applied, including surgery, chemotherapy, and radiotherapy. Recently, the results of a phase 2 study and a large retrospective study confirmed that downstaging from neoadjuvant chemotherapy followed by radical cystectomy could lead to long-term disease control [7, 8]. The MD Anderson group reported that the median overall survival and a disease-specific survival at five years in patients who underwent cystectomy alone were 18.3 months and 20% as compared to 159.5 months and 79% in cystectomy with neoadjuvant chemotherapy; thus, it is possible that surgery with chemotherapy might improve the clinical course [8].

In this case, the patient was diagnosed with invasive baldder carcinoma by the result from urinary cytology and CT/MRI scan findings. Because the bleeding from the tumor couldn not be controlled, he received the radical cystectomy without transurethral resection of baldder tumor. After the surgery, pathological findings revealed a locally advanced SCCB, for the first time, and then adjuvant chemotherapy was carried out.

The chemotherapy administered has often been EP therapy, according to the protocol for primary small cell lung cancer. In particular, cisplatin-based chemotherapy has resulted in improved prognoses compared with a platinum-free regimen [5, 9, 10].

Among the sites of metastasis from bladder carcinoma, the most common site was the lymph node (78%), followed by the liver (38%), lung (36%), and adrenal gland (21%) [11].

Saitoh et al. [12] also reported that metastasis from bladder carcinoma most commonly occurs in the lymph node (61.1%), followed by the lung, liver, and adrenal gland, with the adrenal gland prevalence being 15.5%. But all of these adrenal metastatic cases already had other metastases; this report contained no cases of solitary adrenal metastasis.

Inoue et al. [13] reported a case of unilateral isolated adrenal metastasis from bladder cancer, and it was considered to be extremely rare. The performance of adrenalectomy for solitary adrenal metastasis from bladder carcinoma, especially SCCB as in this case, is limited to a few case reports. Generally, most cases of solitary adrenal metastasis from any cancer are treated with complete surgical resection. The progress and dissemination of diagnostic imaging have enabled the early detection of adrenal metastasis.

In cases which have only a solitary metastasis in the adrenal gland, and in which the primary tumor is controlled well, even in carcinomas that are characterized by aggressive progression in the form of SCC, adrenalectomy may provide a good clinical course.
